# Relationships and gender differences within and between assessments used in Swedish home rehabilitation – a cross-sectional study

**DOI:** 10.1186/s12913-022-08177-x

**Published:** 2022-06-22

**Authors:** Anette Johansson, Cristina Joy Torgé, Sofi Fristedt, Marie Ernsth Bravell

**Affiliations:** 1 Health Care Administration, Jönköping Municipality, Jönköping University, School of Health and Welfare, SE 551 11 Jönköping, Sweden; 2 Jönköping University, School of Health and Welfare, Jönköping, Sweden

**Keywords:** Activities of daily living, Motor function, Self-reported health, Mobility, Older adults, Adults, Gender bias

## Abstract

**Background:**

Home rehabilitation programmes are increasingly implemented in many countries to promote independent living. Home rehabilitation should include a comprehensive assessment of functioning, but the scientific knowledge about the assessment instruments used in this context is limited. The aim of this study was to explore relationships between standardised tests and a self-reported questionnaire used in a home rehabilitation programme. We specifically studied whether there were gender differences within and between assessments.

**Method:**

De-identified data from 302 community-dwelling citizens that participated in a municipal home rehabilitation project in Sweden was analysed. A Mann Whitney test and an independent t-test were used to analyse differences within the following assessment instruments: the Sunnaas Activity of Daily Living Index, the General Motor Function assessment scale and the European Quality of Life Five Dimension Five Level Scale. Spearman’s bivariate correlation test was used to analyse relationships between the instruments, and a Fischer’s Z test was performed to compare the strengths of the correlation coefficients.

**Result:**

Gender differences were found both within and between the assessment instruments. Women were more independent in instrumental activities of daily living than men. The ability to reach down and touch one’s toes while performing personal activities of daily living was stronger for men. There was a difference between men’s self-reported performance of usual activities that included instrumental activities of daily living and the standardised assessment in performing instrumental activities of daily living. The result also showed an overall difference between the self-reported assessment and the standardised test of motor function for the total group.

**Conclusion:**

The results indicate that a comprehensive assessment with the combination of *standardised tests, questionnaires* and *patient-specific instruments* should be considered in a home rehabilitation context in order to capture different dimensions of functioning. Assessment instruments that facilitate a person-centred home rehabilitation supporting personally meaningful activities for both men and women should be applied in daily practice. Further research about gender-biased instruments is needed to facilitate agreement on which specific instruments to use at both individual and organisation levels to promote gender-neutral practice.

**Supplementary Information:**

The online version contains supplementary material available at 10.1186/s12913-022-08177-x.

## Introduction

Home rehabilitation programmes are increasingly used around the world to reduce disability in everyday life due to ageing or health conditions and to promote independent living [1–5]. A home rehabilitation programme is usually a person-centred intervention that includes a comprehensive assessment and is led by multidisciplinary teams [6–8]. As health conditions addressed in home rehabilitation vary, practitioners gain from comprehensive assessments in their daily practice, which allow them to evaluate the progress and effects of rehabilitation interventions [4, 9, 10]. Assessment of functioning is a complex and multifaceted process, since diagnoses, tasks and environments interact and impact on function and activities in different ways [9]. Several gendered factors can also cause differences in health conditions for men and women, especially in old age [11–13], and this could influence assessment outcomes through measurement methods. For example, women are generally at higher risk than men of developing chronic conditions, with subsequent long-term physical impairments and limitations in functioning [14–16]. Self-reported assessments could also be influenced by gender roles, so it is argued that objective measures (i.e. physical performance as observed by staff) are more likely to present gender-neutral assessments [17]. A study found that while there were no significant differences in the physical performance of men and women based on staff assessments, women self-reported a higher incidence of disability in activities of daily living (ADL) and mobility than men over time [18].

To ensure that assessments are valid, reliable and in line with evidence-based practice, standardised assessment instruments should be used [9]. There are three categories of assessment that are commonly used in home rehabilitation programmes to examine a person’s functioning and need for rehabilitation: 1) *standardised examinations and tests* (hereafter *standardised tests*), where staff assess the person’s functioning through observation, using specific pre-determined criteria, 2) *questionnaires*, where standardised self-assessment questions are used, and 3) *patient-specific instruments*, where the person identifies his or her own rehabilitation goals as well as assesses the outcome of the interventions [4, 19]. Unstructured observations are however still commonly used by occupational therapists and physiotherapists [10, 20–22]. Lack of knowledge, confidence and support regarding which assessment instruments to choose in different clinical settings [10, 11] may explain the use of unstructured rather than structured assessments. Moreover, there is no overall agreement on which specific instruments that should be used within home rehabilitation programmes [6, 21].

When using a range of instruments, it is vital to know how the instruments relate to each other and the different aspects covered by each. Gender differences in functioning and possible differences between self-reported and standardised tests need to be considered when planning which assessment instruments and evaluation methods to use in home rehabilitation practice [15, 16, 21, 23]. In this study, we aimed to explore relationships between *standardised tests* and a *questionnaire* used in a municipal home rehabilitation context, and we specifically studied whether there were gender differences within and between assessments.

## Method

### Data collection

A home rehabilitation project was undertaken in a Swedish municipality with approximately 140,000 citizens during the period 2015–2020. In this particular study, we used previously collected data from this project. The Swedish Ethical Review Authority (Dnr 2019–00706) approved the research study, and access to de-identified data was granted by the municipality in line with Swedish laws and regulations [24].

### The home rehabilitation participants

The intervention was offered to adult citizens living in a selected geographical area within the municipality (mainly urban or suburban areas of approximately 47,000 inhabitants). Eligible participants were adults living in ordinary homes, in all ages, with a broad range of health conditions requiring rehabilitation. Enrolment was preceded by a dialogue based on the person’s health and functioning, wishes and needs, and willingness to undergo intensive rehabilitation. A more detailed description of the home rehabilitation process is described elsewhere [25, 26]. Of the 311 persons who agreed to participate in the intervention, nine persons had no collected data (unknown reason *n* = 4, declined *n* = 3, and worsening health *n* = 2). Consequently, a total of 302 participants (197 women and 105 men) were included in the analyses for this study (Table 2).

### The home rehabilitation intervention

The intervention involved up to 12 weeks of training in the person’s own home and neighbourhood, led by an interdisciplinary team of occupational therapists (OTs), physiotherapists (PTs) and rehabilitation assistants. The participants formulated their own goals together with the rehabilitation staff and, if applicate, with their next of kin. The goals included personal ADL (PADL), instrumental ADL (IADL), social activities and hobbies. The intervention also involved close collaboration with other caregivers such as home care staff and registered nurses.

### Assessment methods

The home rehabilitation team used a battery of pre-determined assessments to collect data at the start and at the end of the intervention, and at a follow-up 2 months after the intervention period. Participant characteristics such as age (years), gender (male/female), living situation (living alone/cohabiting) and main reasons for home rehabilitation were also collected. The data was collected by the OTs and PTs who also carried out the home rehabilitation intervention. The program included clear and structured routines for data collection and data handling. In this particular study, we use data collected at start of the home rehabilitation intervention. We specifically analysed two *standardised test* instruments concerning ADL and motor function and one *questionnaire* concerning self-rated health. To structure the participants’ main reasons for rehabilitation, we classified these into seven categories using information on current diagnosis, medical condition, and affected body part (e.g., mobility difficulties due to hip fracture). The categories were: *condition of the circulatory and respiratory systems, mobility limitations including fall risk, multimorbidity and/or frailty*, *neurological conditions excl. stroke*, *orthopaedic conditions upper extremities and spine*, *orthopaedic conditions lower extremities and pelvis* and *stroke* (a fuller description is presented elsewhere [25]).

#### Standardised tests used

##### Sunnaas ADL index

The Sunnaas Activity of Daily Living Index (Sunnaas ADL index) examines the person’s functioning in daily activities [27]. The Sunnaas ADL index contains ratings of 12 defined daily activities, involving eight PADL and four IADL. The level of independence in performing these tasks was scored as follows: 3 = completely independent, 2 = independent but requires aids or adapted environment, 1 = partly dependent on another person and 0 = completely dependent on another person [28]. The Sunnaas ADL index is considered reliable and valid [28–30], and the scoring was done by OTs in the home rehabilitation team.

##### General motor function

The General Motor Function (GMF) assessment scale includes 11 mobility functions and 10 upper limb functions [31] and is summarised in three different subscales: function-related dependence (hereafter dependence), pain, and insecurity. Degrees of dependence were assessed on a two-point scale for some functions (0 = independent, 1 = requires the help of one person/unable to manage) [31] or on a three-point scale for other functions (0 = independent, 1 = help of one person 2 = help of two persons/unable to manage) [31] (Table 3). As the GMF subscales pain and insecurity were based on participants’ self-reported experience rather than standardised observation, these scales were excluded from analyses. The GMF has shown good validity [32, 33], sensitivity [32] and reliability [31], and the assessment was completed by PTs in the home rehabilitation team.

#### Questionnaire used

##### European quality of life five dimension five level scale

Self-rated health was measured using the Swedish version of the European Quality of Life Five Dimension Five Level Scale (EQ-5D-5 L) [34]. The instrument contains two parts: a questionnaire rating five dimensions (*mobility*, *personal care*, *usual activities*, *pain/discomfort*, *anxiety/depression*) and a visual analogue scale (EQ VAS). Utilising a Likert scale with five levels (1 = no problems, 5 = extreme problems), the participants were asked to choose the most appropriate statement in each of the five dimensions. In EQ VAS, participants assessed their present day health status using a vertical visual analogue scale ranging from 0 (“the worst imaginable health”) to 100 (“the best imaginable health”) [34]. The EQ-5D-5 L has demonstrated satisfactory reliability, validity and responsiveness in older adults [35]. The home rehabilitation team asked participants to complete the EQ-5D-5 L form and return it by mail in a pre-stamped envelope addressed to the municipality development unit.

### Analyses of relations within and between assessments

Gender differences in demographics were analysed with an independent t-test and a Pearson Chi square test. Gender differences within instruments were analysed using a Mann Whitney test for variables at ordinal level (Sunnaas ADL index, GMF and EQ-5D-5 L dimensions) and an independent t-test for the normally distributed variable at interval level (EQ VAS). The Mann Whitney was chosen due to the ordinal level of the data, no clear normal distribution in the variables and to the independent group design. As we found gender differences in Sunnaas IADL’s *cooking* and *housework* in the total group, we did a subgroup analysis to identify whether life situation could explain these differences among single-living or partnered men and women, as functioning in household activities could be associated with gender norms in performing these tasks.

We further compared variables from the different assessment instruments that corresponded to similar or relevant aspects of functioning (Table 1), based on literature describing them [27, 31, 34]. Using our professional knowledge of the field, the authors discussed which variables to compare until consensus was reached. As no variables from the Sunnaas ADL index covered aspects similar to the EQ-5D-5 L dimension’s pain/discomfort and anxiety/depression, these were excluded from the correlation analysis. The variables corresponding to the left part of the body were excluded from the correlation analyses as no major differences in mean and standard deviation between right and left hand/arm variables in the GMF dependency scale were found.

**Table 1:** Variables compared from the EQ-5D-5 L, Sunnaas ADL index and GMF dependence subscale

EQ-5D-5 L	Sunnaas ADL	GMF dependence
Mobility *(walking about)*	Indoor mobility *(mobility*^a^ *at home and at work)* Outdoor mobility *(*e.g.*, go outdoors, use transportation, do errands, visit family and friends, shopping)*	Mobility functions Transfer indoors * (mobility*^a^ *10 m)* Climb stairs * (up/down 7 steps)* Transfer outdoors * (mobility*^a^ *25 m)*
Self-care *(washing or dressing)*	Dressing/undressing *(put on clothes,* e.g.*, socks, pants, outer garments)* Grooming *(daily hygiene,* e.g.*, brush teeth, comb hair)* Bath/shower *(including using taps, drying afterwards)*	Mobility functions Touch big toe * (sitting in chair)* Stand up from a sitting position Stand more than 10 seconds Upper limb functions Move hand to mouth Move hand to head * (to highest part of head)* Move hand on back * (to upper part of sacrum)* Greeting grip Pinch grip
Usual activities *(work, studies, housework, family, or leisure activities)*	Cooking *(*e.g.*, prepare a simple hot meal, open food wrapping)* Housework *(*e.g.*, cleaning, laundry, dishwashing)* Outdoor mobility *(*e.g.*, go outdoors, use transportation, do errands, visit family and friends, cultural events)*	Mobility functions Touch big toe * (sitting in chair)* Stand up from a sitting position Stand more than 10 seconds Transfer indoors * (mobility*^a^ *10 m)* Transfer outdoors * (mobility*^a^ *25 m)* Upper limb functions Move hand to mouth Move hand to head * (to highest part of head)* Move hand on back * (to upper part of sacrum)* Greeting grip Pinch grip

^a^mobility with or without assistive devices (e.g., rollator, wheelchair). Definitions in parentheses

To analyse relationships between the compared variables, a Spearman’s bivariate correlation analysis was performed, with an approach similar to a previous study [12]. Firstly, the compared variables in the Sunnaas ADL index and the GMF dependence subscale were analysed for the total group, then separately for men and women. Secondly, the compared variables in the EQ-5D-5 L, Sunnaas ADL index, and GMF dependence subscale were analysed for the total group, then separately for men and women. Correlation values between 0 to .29 were considered to be weak, .3 to .69 to be moderate and .7 and 1 to be strong [36]. The Bonferroni correction test was used to adjust for multiple testing. Fisher’s Z test was performed to compare the strength of the correlation coefficient between men and women in the bivariate correlation analysis. *P* values < 0.05 were considered statistically significant. Statistical analyses were performed with IBM SPSS Statistic version 26.

## Results

### Descriptive

The mean age of the participants was 80 years and most were women, see Table 2. A majority of the women lived alone whereas a majority of men cohabited with someone. The most frequent main reasons for home rehabilitation in the total group were *multimorbidity and/or frailty* and *orthopaedic conditions (lower extremities and pelvic)* (Table 2). More men experienced *neurological conditions (excl. stroke)* while more women experienced different *orthopaedic conditions* (Table 2).

**Table 2:** Description of participants’ characteristics and group comparisons

Characteristics	Men (*n* = 105)	Women (*n* = 197)	Total (*n* = 302)	*t*	CI 95%
Age in years; mean (SD), min-max	78.9 (9.3), 49–99	80.1 (9.2), 39–96	79.7 (9.2), 39–99	−1.152	−3.48 - .910
	**n (%)**	**n (%)**	**n (%)**	***×***^***2***^	***P -*value**
Living situation;				20.86	**> .001**
Living alone	35 (33.3)	120 (60.9)	155 (51.3)		
Cohabiting	70 (66.7)	77 (39.1)	147 (48.7)		
Main reasons for home rehabilitation	**n (%)**	**n (%)**	**n (%)**		
Condition of circulatory and respiratory systems	10 (9.5)	18 (9.1)	28 (9.3)	.012	.912
Mobility limitations including fall risk (condition not specified)	8 (7.6)	13 (6.6)	21 (7.0)	.110	.740
Multimorbidity and/or frailty	33 (31.4)	44 (22.3)	77 (25.5)	2.98	.84
Neurological conditions (excl. stroke)	8 (7.6)	3 (1.5)	11 (3.6)	7.25	**.007**
Orthopaedic conditions (upper extremities and spine)	6 (5.7)	35 (17.8)	41 (13.6)	8.48	**.004**
Orthopaedic conditions (lower extremities and pelvis)	16 (15.2)	56 (28.4)	72 (23.8)	6.56	**.010**
Stroke (acute and post)	24 (22.9)	28 (14.2)	52 (17.2)	3.59	.058

*Abbreviations*: *SD* Standard deviation, *CI* Confidence interval, *t* Independent t-test, *x*^*2*^ Pearson Chi square test

### Gender differences within the assessment instruments

There were no statistically significant differences between men and women in self-reported health, as displayed in Table 3. Women were significantly more independent in the Sunnaas IADLs’ *cooking* and *housework* (Table 3). In our additional Mann Whitney analysis, we found no differences between men and women living alone regarding the Sunnaas IADL *cooking* (*U* = 1931, *p =* .441), and *housework* (*U* = 1853.5, *p* = .201). Comparing cohabiting men and women, the women were significantly more independent than men in Sunnaas IADL *cooking* (*U* = 1977.5, *p* = .003) but not in Sunnaas IADL *housework* (*U* = 2382.5, *p* = .169). Men were significantly more independent in GMF dependence *climb stairs up/down 7 steps,* and women were significantly more independent in *greeting grip with left hand* (Table 3).

**Table 3:** Differences between genders within the assessment instrument

Variables	Men			Women					
EQ-5D-5 L	**n**	**mean (SD)**		**n**	**mean (SD)**		**CI 95%**	***t***	***p*-value**
EQ VAS (1–100)^a^	71	53.77 (20.0)		147	49.97 (19.7)		−1.8-9.4	1.33	.186
Dimensions^b^	**n**	**md**	**interquartile range**	**n**	**md**	**interquartile range**	***U***		***p*-value**
mobility (1–5)	72	3	2–4	147	3	2–4	5084.5		.625
self-care (1–5)	72	3	2–3	147	3	2–3	5125.5		.693
usual activities (1–5)	72	4	3–4	148	4	3–4	5110.0		.608
pain/discomfort (1–5)	72	3	2–3	147	3	3–3	4692.5		.137
anxiety/depression (1–5)	72	2	1–2.75	148	2	1–3	5016.0		.458
Sunnaas ADL index^c^									
eating (0–3)	105	3	3–3	197	3	3–3	9837.0		.196
continence (0–3)	105	3	2–3	197	3	2–3	10,055.5		.653
indoor mobility (0–3)	105	2	2–2	197	2	2–2	10,245.0		.871
toilet management (0–3)	105	2	2–3	197	2	2–3	10,217.5		.847
transfer (0–3)	105	2	2–3	197	2	2–3	10,226.0		.860
dressing/undressing (0–3)	105	2	1–3	197	2	1–3	10,109.5		.732
grooming (0–3)	105	3	2–3	197	3	2–3	9687.5		.309
cooking (0–3)	105	1	0–2	197	2	1–2	7288.5		**< .001**
bath/shower (0–3)	105	1	1–2	197	1	1–2	10,242.0		.877
housework (0–3)	105	0	0–1	197	1	0–1	8315.5		**.002**
outdoor mobility (0–3)	105	1	1–1	197	1	1–1	9753.5		.363
communication (0–3)	105	3	3–3	197	3	3–3	9896.0		.334
GMF dependence^d^ Mobility functions									
turn around when lying in bed (0–2)	97	0	0–0	181	0	0–0	13,347.0		.553
sit up from recumbent position (0–2)	98	0	0–0	181	0	0–0	8495.5		.173
lie down from a sitting position (0–2)	98	0	0–0	181	0	0–0	8585.5		.288
transfer from bed to chair (0–2)	98	0	0–0	181	0	0–0	8473.0		.222
touch left big toe (0–1)	97	0	0–0	176	0	0–0	8383.0		.717
touch right big toe (0–1)	97	0	0–0	174	0	0–0	7814.0		.138
stand up from a sitting position (0–2)	98	0	0–0	181	0	0–0	8369.0		.148
stand more than 10 seconds (0–2)	98	0	0–0	181	0	0–0	8827.0		.886
transfer indoors 10 m (0–2)	98	0	0–0	181	0	0–0	8262.5		.057
climb stairs up/down 7 steps (0–2)	98	0	0–1	172	1	0–1	7224.0		**.036**
transfer outdoors 25 m (0–2)	98	1	0–1	179	1	0–1	8586.5		.750
Upper limb functions									
move left hand to mouth (0–1)	98	0	0–0	181	0	0–0	8612.5		.193
move right hand to mouth (0–1)	98	0	0–0	181	0	0–0	8805.0		.713
move left hand to head (0–1)	98	0	0–0	181	0	0–0	8578.5		.232
move right hand to head (0–1)	99	0	0–0	181	0	0–0	8891.5		.829
move left hand on back (0–1)	99	0	0–0	181	0	0–0	8459		.090
move right hand on back (0–1)	99	0	0–0	181	0	0–0	8702.0		.421
greeting grip with left hand (0–2)	98	0	0–0	181	0	0–0	8313.5		**.037**
greeting grip with right hand (0–2)	98	0	0–0	181	0	0–0	8734.5		.614
pinch grip with left hand (0–2)	98	0	0–0	181	0	0–0	8802.0		.746
pinch grip with right hand (0–2)	98	0	0–0	181	0	0–0	8788.0		.730

*Abbreviations*: *SD* Standard deviation, *CI* Confidence interval, *md* median, *t* independent t-test, *U* Mann Whitney test. Notes: ^a^better perceived health shows in higher scores; ^b^fewer problems show in lower scores; ^c^higher degree of independence shows in higher scores; ^d^higher degree of independence shows in lower scores

### Correlations between the two standardised test instruments

#### Analysis of the total group

**Fig. 1:**
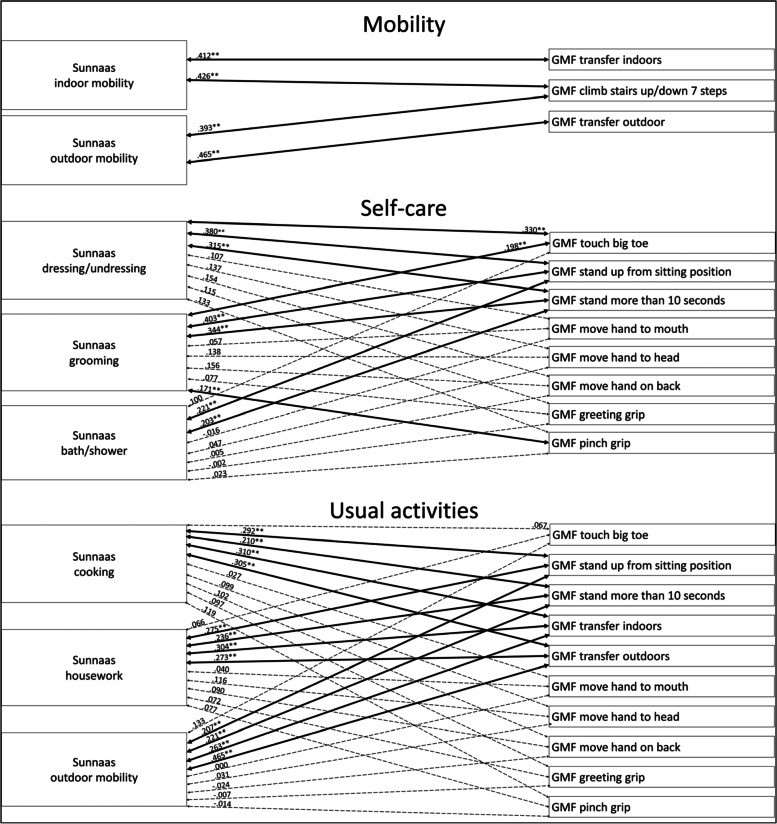
Correlations between Sunnaas ADL and GMF dependence variables with the total group. Notes: thick bold line = *p* < .01, thin bold line = *p* < .05, dotted line = not statistically significant

Considering mobility within the total group, the Sunnaas ADL variables correlated significantly with the GMF mobility function variables, with a moderate strength (Fig. 1). For self-care, the Sunnaas PADL *dressing/undressing*, *grooming* and *bath/shower* correlated significantly with most GMF mobility function variables, with a weak to moderate strength (Fig. 1)*.* The Sunnaas ADL variables did not correlate significantly with the GMF upper limb functions, except for Sunnaas PADL *grooming* with GMF dependence *pinch grip*, with a weak strength (Fig. 1). Regarding usual activities, the Sunnaas ADL variables correlated significantly with most GMF mobility functions, with a weak to moderate strength (Fig. 1)*.* There were no statistically significant correlations between the Sunnaas ADL variables and the GMF upper limb functions (Fig. 1).

#### Analysis of men in the group

**Fig. 2:**
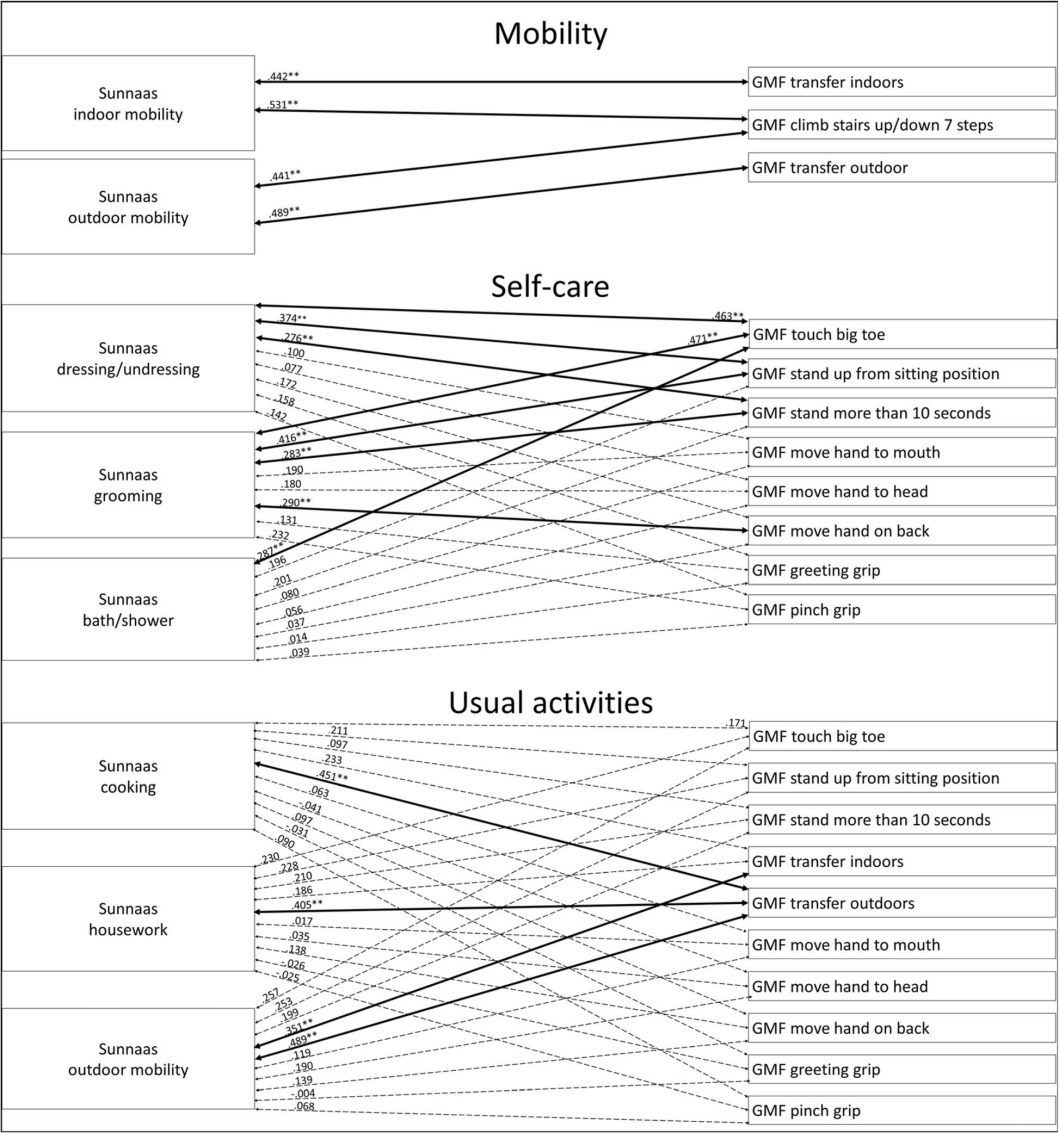
Correlations between Sunnas ADL and GMF dependence variables with men. Notes: thick bold line = *p* < .01, thin bold line = *p* < .05, dotted line = not statstically significant

Considering mobility among the men in the group, the Sunnaas ADL variables correlated significantly with the GMF mobility function variables, with a moderate strength (Fig. 2). For self-care, the Sunnaas PADL *dressing/undressing* and *grooming* correlated significantly with the GMF mobility function variables, with a weak to moderate strength (Fig. 2). The Sunnaas PADL *bath/shower* correlated significantly with GMF dependence *touch big toe.* The Sunnaas PADL *grooming* correlated significantly with GMF dependence *move hand on back*, with a weak strength; otherwise, there were no statistically significant correlations between the Sunnaas ADL variables and the GMF upper limb functions (Fig. 2). Regarding usual activities, the Sunnaas IADL *cooking, housework* and *outdoor mobility* correlated significantly with GMF dependence *transfer outdoors*, with a moderate strength, where the Sunnaas IADL *outdoor mobility* also correlated significantly with GMF dependence *transfer indoors* (Fig. 2). There were no statistically significant correlations between the Sunnaas ADL variables and the GMF upper limb functions (Fig. 2).

#### Analysis of women in the group

**Fig. 3:**
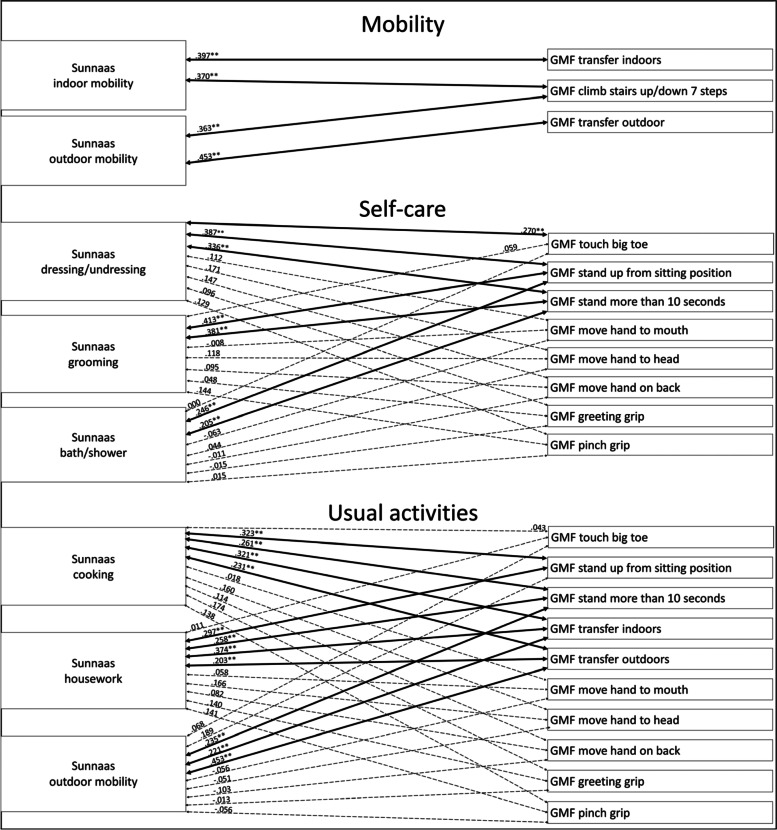
Correlations between the Sunnaas ADL and GMF dependence variables with women. Notes: thick bold line = *p* < .01, thin bold line = *p* < .05, dotted line = not statistically significant

Considering mobility among the women in the group, the Sunnaas ADL variables correlated significantly with the GMF mobility function variables, with a moderate strength (Fig. 3). For self-care, the Sunnaas ADL variables correlated significantly with most GMF mobility functions, with a weak to moderate strength (Fig. 3). There were no statistically significant correlations between the Sunnaas ADL variables and the GMF upper limb functions (Fig. 3). Regarding usual activities, the Sunnaas ADL variables correlated significantly with most GMF mobility function variables, with weak to moderate strength (Fig. 3). There were no statistically significant correlations between the Sunnaas ADL variables and the GMF upper limb functions (Fig. 3).

The analyses shows that there are some differences in the patterns between men’s and women’s assessments. However, the statistically significant differences in men’s and women’s correlation coefficients were between the Sunnaas PADL *dressing/undressing* and *grooming* and GMF dependence *touch big toe*, and between the Sunnaas IADL *cooking* and GMF dependence *transfer outdoors.* Both correlations were stronger for men (see Additional file 1, Fischer’s Z test).

### Correlations between self-reported assessment and assessment with a standardised test

#### Analysis of the total group

**Fig. 4:**
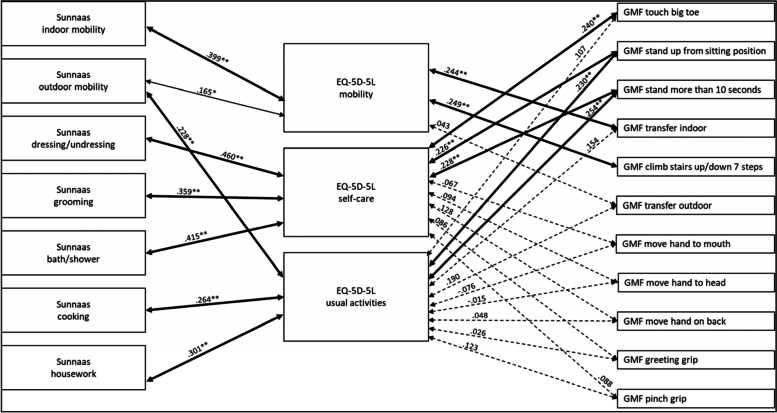
Correlations between grouped variables of the Sunnaas ADL index, EQ-5D-5 L and GMF dependence subscale with the total group. Notes: thick bold line = *p* < .01, thin bold line = *p* < .05, dotted line = not statistically significant

The results for the total group showed statistically significant correlations between the compared variables (Fig. 4) regarding the Sunnaas ADL and the EQ-5D-5 L, with a weak to moderate strength. The EQ-5D-5 L dimension *self-care* correlated significantly with compared GMF mobility functions, with a weak strength, whereas the EQ-5D-5 L dimensions *mobility* and *usual activities* correlated significantly with some of them (Fig. 4). There were no statistically significant correlations between the EQ-5D-5 L dimensions *self-care* and *usual activities,* and the GMF upper limb functions (Fig. 4).

#### Analysis of men in the group

**Fig. 5:**
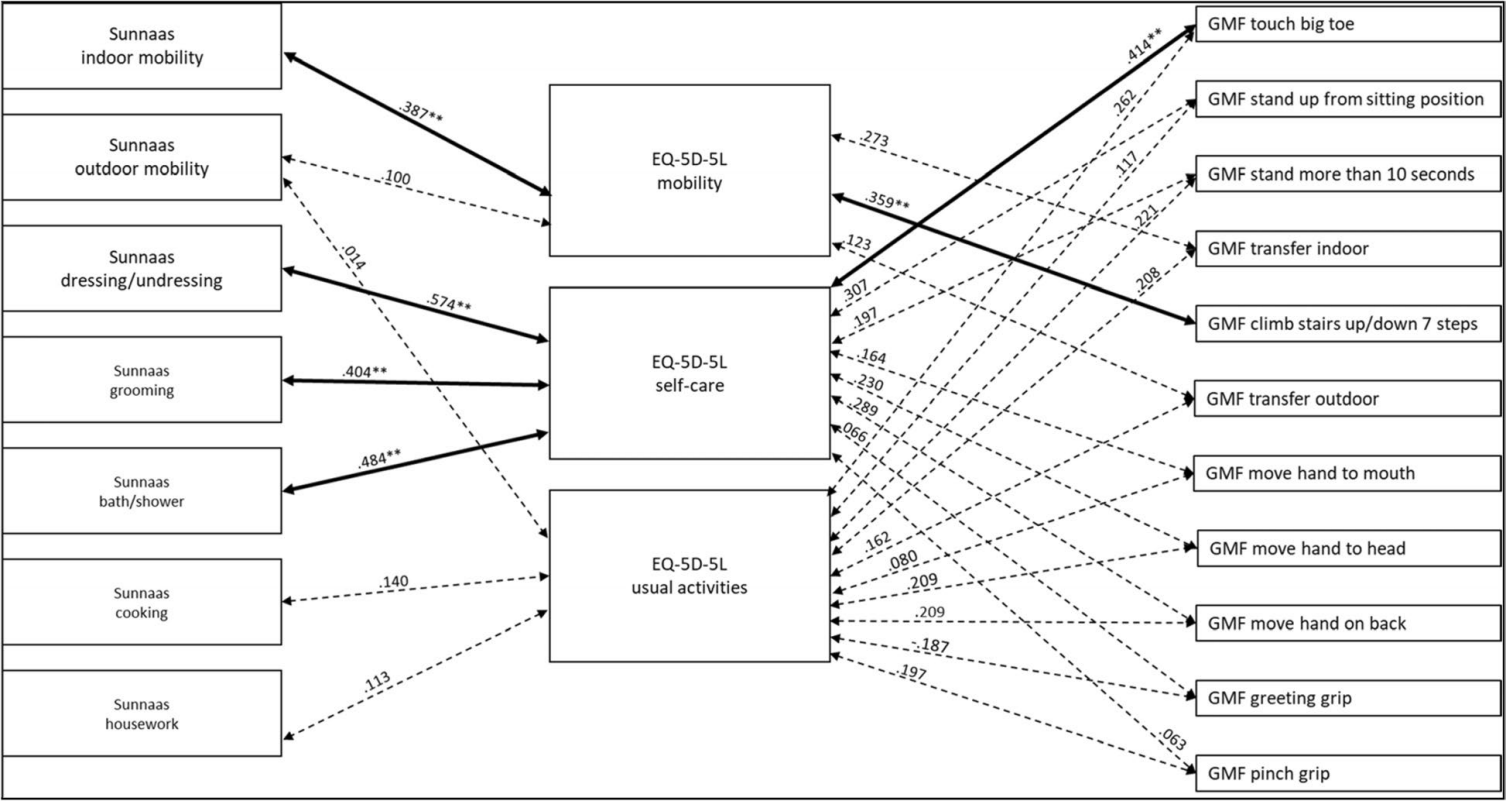
Correlations between compared variables of the Sunnaas ADL index, EQ-5D-5 L and GMF dependence subscale with men. Notes: thick bold line = *p* < .01, thin bold line = *p* < .05, dotted line = not statistically significant

When looking only at men in the group, the EQ-5D-5 L dimension *usual activities* did not correlated significantly with any of the GMF variables that were compared (Fig. 5). The EQ-5D-5 L dimension *self-care* correlated significantly with the compared Sunnaas ADL variables and with GMF dependence *touch big toe*, with a moderate strength. The EQ-5D-5 L dimension *self-care* did not correlated significantly with GMF upper limb functions (Fig. 5)*.* The EQ-5D-5 L dimension *mobility* significantly correlated with Sunnaas PADL *indoor mobility*, with a moderate strength, and with GMF dependence *climb stairs up/down 7 steps*, with a moderate strength (Fig. 5).

#### Analysis of women in the group

**Fig. 6:**
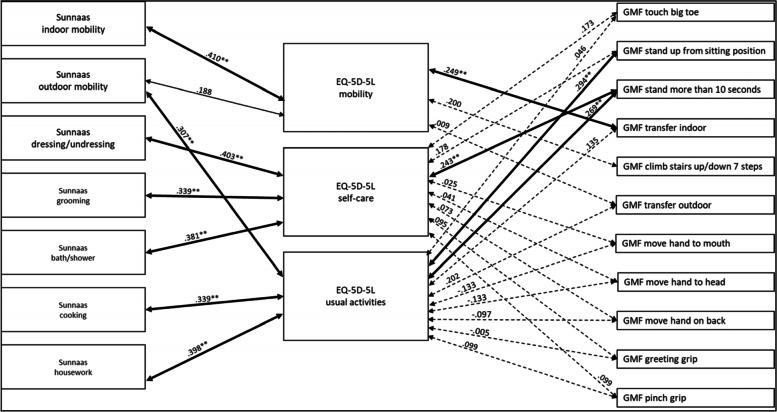
Correlations between compared variables of the Sunnaas ADL index, EQ-5D-5 L and GMF dependence subscale with women. Notes: thick bold line = *p* < .01, thin bold line = *p* < .05, dotted line = not statistically significant

Looking at the women in the group, there were statistically significant correlations between the Sunnaas ADL and the EQ-5D-5 L variables, with weak to moderate strength (Fig. 6). The EQ-5D-5 L dimension *mobility, self-care* and *usual activities* correlated significantly with some of the compared GMF mobility functions, with a weak strength (Fig. 6). There were no statistically significant correlations between the EQ-5D-5 L dimensions *self-care* and *usual activities,* and the GMF upper limb functions (Fig. 6).

The analyses show gender differences, for example between the EQ-5D-5 L dimension *usual activities* and Sunnaas ADL, where there was a statistically significant correlation for women but not for men. The difference was statistically significant regarding the EQ-5D-5 L dimension *usual activities* and Sunnaas *housework* and *outdoor mobility* (see Additional file 2, Fischer’s Z test).

## Discussion

The aim of this study was to investigate relationships between assessments from *standardised tests* and a *questionnaire* and to determine if there were gender differences, both within and between the assessment instruments used. We found that women were assessed to be more independent regarding the IADLs of cooking and housework compared to men. We argue that this may not only be due to differences in functioning but may also be an effect of traditional gender roles. Other studies likewise show that women are still more often engaged in these IADLs than their male partners [37, 38]. The fact that we found no such gender differences between men and women living alone and that cohabiting women were more independent in IADL cooking, could also support this explanation. In our study, like previous studies [38–40], the majority of men lived with someone (for example a wife taking care of cooking) while the female participants mostly lived alone. Bias could therefore have played a role in the score setting, if women were assessed by their functioning and men by their knowledge or skills in certain household activities [17]. A similar finding was observed in a study that concluded that men and women were not equivalently observed on IADLs [41]. The ADL test used in the intervention did not include traditional “male chores” (e.g., car care and minor household repairs). Our findings call for using instruments that target a wider range of daily chores, to avoid gender-biased results in assessments of ADL ability, and to support gender-equal practice in line with health and social service policies [42]. It is important to take in consideration that gender differences in household activities are expected to decrease through generations. Further research should therefore analyse whether different age cohorts lead to different results.

Another interesting result in our study is that the staff’s assessment of IADL was consistent with women’s self-assessment in the EQ-5D-5 L dimension *usual activities* but not the men’s self-assessments. This may indicate that men thought of other activities outside IADL when responding to the EQ-5D-5 L dimension *usual activities*. For example, a previous study found differences in how older persons use their mobility outside the home. They found that men engaged in more leisure activities such as sports, whereas women were more engaged in IADL [37]. It has also been suggested that older women find household activities more interesting and meaningful than men do [38]. What typical “meaningful activities” can be may be gendered. Thus, the gendered dimensions of the IADLs measured in *standardised tests* highlight the importance of individual goal-setting, for example with *patient-specific instruments*, where goals related to the person’s own context also are likely to be identified in the home rather than in the clinical context. For example, Canadian Occupational Performance Measure (COPM) is a *patient-specific instrument* relevant to use in home rehabilitation programs that helps the person to identify meaningful, everyday activities during the goal-setting process [4].

We found that correlations between the three assessment instruments were weak to moderate. For example, the concordance between Sunnaas ADL and GMF upper limb functions was particularly low, and with the Bonferroni correction test most of the significant relationships disappeared. An explanation could be that the participants had physical impairments but still managed to be independent in ADL. If so, this indicates that a performance-based standardised test of physical function poorly predicts the performance of more complex ADL situations [12]. In addition, we found a gender difference in motor functioning in reaching down and touching one’s toes during PADL and climbing stairs, to the men’s advantage. This could be explained by the fact that most women in our study suffered from orthopaedic conditions and multimorbidity, which also included long-term chronic diseases affecting their motor functioning to a greater extent than for men [15, 16]. When it comes to the two standardised tests measuring ADL and motor functions, the mobility variables tend to overlap each other. A suggestion for the home rehabilitation team could therefore be to choose broad standardised tests that covers more than just mobility aspects.

We found no differences in how men and women self-report their perceived health in three of the EQ-5D-5 L dimensions. Our results contrast with the notion that women generally over-report and men under-report health problems, but is in line with a recent study suggesting otherwise [43]. However, we found a difference between EQ-5D-5 L and the standardised assessment with GMF, with a few weak statistically significant correlations between them. This result is in line with previous studies which suggest that physical functioning measured by staff on the one hand, and self-reported measures of physical functioning on the other hand do not measure the same construct [12, 13]. Another possible explanation for the difference could be that self-reported estimates are influenced by emotional, psychological and environmental factors that are not present in standardised tests assessing more specific aspects of functioning [13]. Additionally, not all physical impairments lead to functional limitations or experienced disability. There is thus an added value to measuring people’s subjective experience of health besides objective measurements of function and activity. Our results suggest the importance of combining all the three types of assessments (standardised examinations, self-assessment questionnaires and patient-specific instruments) in home rehabilitation, to ensure complementary information is obtained. This would provide a wider overall picture of the person’s situation [4] and to capture functional improvements that are not self-reported [11].

In our study we have analysed data from a real-life setting with a comprehensive structured assessment implemented in everyday practice. However, as previously described, there are factors that hinder the use of standardised assessment instruments in practice [10, 11] and this needs to be addressed when implementing home rehabilitation programs. In our study, we have also analysed data from assessments instruments used by an interdisciplinary team. Together, the instruments target body functions, activity, and participation in line with The Geriatric ICF Core Set. The Geriatric ICF Core Set is developed from WHO’s International Classification of Functioning, Disability and Health (ICF) to reflect the most relevant health-related problems of community-dwelling older adults [44]. Home rehabilitation programs often also include assessments from other health care/care providers. Thus, to avoid fragmented care, The Geriatric ICF Core Set could be used as a framework to unify the assessments of older adults’ functioning and disability as their health is a multidimensional construct [44]. Further research on the associations between The Geriatric ICF Core Set and instruments used in home rehabilitation settings is needed.

### Strength and limitations

We scientifically analysed clinical data from a real-life setting. Accordingly, the results of our study have relevance to practice. Our results, with a sample varying in age (although the majority were older adults), gender, living situations and health conditions, can be externally generalised to similar contexts. However, one limitation of real-life data is the difficulty to control for other explanations affecting the result. Another limitation of our study is that that the participants were evaluated by different assessors. Nevertheless, the assessors were trained professionals and had ongoing discussions on how to use the instruments, for consensus and to ensure the reliability of the data. Our study is similar to a previous study [12] that studied agreements between different categories of assessment instruments. However, we are aware that the study is not a method study and have therefore chosen the analysis methods that are in line with our purpose.

## Conclusion

The use of comprehensive assessments, especially when gender aspects are considered, allows subtle differences in functional abilities in the older population to be better captured [4, 40]. Our result indicates that *standardised tests* alone do not capture all dimensions of functioning. The differences between the self-reported *questionnaire* and *standardised test* outcomes shows the importance of combining these categories of assessments instruments to capture different perspectives. To obtain a person-centred intervention focusing on meaningful activities a *patient-specific instrument* should also be included in the assessment battery. Further research is needed on which specific instruments within the categories to use in home rehabilitation programs for a gender-neutral practice.

## Supplementary Information

**Additional file 1.** Fischers Z-test between men and women correlation coefficient Sunnaas ADL and GMF.

**Additional file 2.** Fischers Z-test between men and women correlation coefficient EQ5D, Sunnaas ADL and GMF.

## Data Availability

The data that support the findings of this study were collected within a project run by the municipality of Jönköping, Sweden. Strong restrictions apply to the availability of these data, which were used under license for the current study, and so are not publicly available. Data used for this study could however be available from the authors upon reasonable request and if permission from the municipality of Jönköping is granted.

## Abbreviations

ADL: Activities of daily living
Sunnaas ADL index: Sunnaas Activity of Daily Living Index
PADL: Personal activities of daily living
IADL: Instrumental activities of daily living
GMF: General Motor Function
EQ-5D-5 L: European Quality of Life Five Dimension Five Level Scale
EQ VAS: European Quality of Life visual analogue scale
ICF: International Classification of Functioning, Disability and Health
