# Supplementary material for: Relationships and gender differences within and between assessments used in Swedish home rehabilitation – a cross-sectional study
Source: BMC Health Serv Res. 2022 Jun 22;22:807. doi: 10.1186/s12913-022-08177-x (PMC9215038; doi:10.1186/s12913-022-08177-x)
Supplement: Supplementary file 1 — Additional file 1. Fischers Z-test between men and women correlation coefficient Sunnaas ADL and GMF. [file 12913_2022_8177_MOESM1_ESM.docx]

Additional file 1. Fischers Z-test between men and women correlation coefficient Sunnaas ADL and GMF

| variables | r1 | n1 | r2 | n2 | zl | prob.valueα |
| --- | --- | --- | --- | --- | --- | --- |
| indoor mobility*transfer indoors | .442 | 98 | .397 | 181 | 0.43 | 0.67 |
| indoor mobility*climb stairs | .531 | 97 | .370 | 171 | 1.58 | 0.11 |
|  |  |  |  |  |  |  |
| outdoor mobility*climb stairs | .441 | 97 | .363 | 171 | 0.72 | 0.47 |
| outdoor mobility*transfer outdoor | .489 | 98 | .453 | 178 | 0.36 | 0.72 |
|  |  |  |  |  |  |  |
| dressing*touch big toe | .463 | 96 | .270 | 173 | 1.74 | 0.08 |
| dressing*stand up | .374 | 98 | .387 | 181 | -0.12 | 0.90 |
| dressing*stand 10 sec. | .276 | 98 | .336 | 181 | -0.52 | 0.60 |
| dressing*move hand to mouth | .100 | 98 | .112 | 181 | -0.10 | 0.92 |
| dressing*move hand to head | .077 | 98 | .171 | 180 | -0.75 | 0.45 |
| dressing*move hand to back | .172 | 98 | .147 | 180 | 0.20 | 0.84 |
| dressing*greeting grip | .158 | 98 | .096 | 181 | 0.50 | 0.62 |
| dressing*pinch grip | .142 | 98 | .129 | 181 | 0.10 | 0.92 |
|  |  |  |  |  |  |  |
| grooming*touch big toe | .471 | 96 | .059 | 173 | **3.51** | **<0.001** |
| grooming*stand up | .416 | 98 | .413 | 181 | 0.03 | 0.98 |
| grooming*stand 10 sec. | .283 | 98 | .381 | 181 | -0.87 | 0.39 |
| grooming*right hand mouth | .190 | 98 | -.008 | 181 | 1.58 | 0.11 |
| grooming*move hand to head | .180 | 98 | .118 | 180 | 0.50 | 0.62 |
| grooming*move hand to back | .290 | 98 | .095 | 180 | 1.60 | 0.11 |
| grooming*greeting grip | .131 | 98 | .048 | 181 | 0.66 | 0.51 |
| grooming*pinch grip | .232 | 98 | .144 | 181 | 0.72 | 0.47 |
|  |  |  |  |  |  |  |
| bath/shower*touch big toe | .287 | 96 | .000 | 173 | **2.29** | **0.02** |
| bath/shower*stand up | .196 | 98 | .246 | 181 | -0.41 | 0.68 |
| bath/shower*stand 10 sec. | .201 | 98 | .205 | 181 | -0.03 | 0.97 |
| bath/shower*move hand to mouth | .080 | 98 | -.063 | 181 | 1.13 | 0.26 |
| bath/shower*move hand to head | .056 | 98 | .044 | 180 | 0.09 | 0.92 |
| bath/shower*move hand to back | .037 | 98 | -.011 | 180 | 0.38 | 0.71 |
| bath/shower*greeting grip | .014 | 98 | -.015 | 181 | 0.23 | 0.82 |
| bath/shower*pinch grip | .039 | 98 | .015 | 181 | 0.19 | 0.85 |
|  |  |  |  |  |  |  |
| cooking*touch big toe | .171 | 96 | .043 | 173 | 1.01 | 0.31 |
| cooking *stand up | .211 | 98 | .323 | 181 | -0.95 | 0.34 |
| cooking *stand 10 sec. | .097 | 98 | .261 | 181 | -1.34 | 0.18 |
| cooking *transfer indoor | .233 | 98 | .321 | 181 | -0.75 | 0.45 |
| cooking *transfer outdoor | .451 | 98 | .231 | 178 | **1.97** | **0.05** |
| cooking *move hand to mouth | .063 | 98 | .018 | 181 | 0.35 | 0.72 |
| cooking *move hand to head | -.041 | 98 | .160 | 180 | -1.59 | 0.11 |
| cooking *move hand to back | .097 | 98 | .114 | 180 | -0.14 | 0.89 |
| cooking *greeting grip | -.031 | 98 | .174 | 181 | -1.63 | 0.10 |
| cooking *pinch grip | .090 | 98 | .138 | 181 | -0.38 | 0.70 |
|  |  |  |  |  |  |  |
| housework*touch big toe | .230 | 96 | .011 | 173 | 1.73 | 0.08 |
| housework*stand up | .228 | 98 | .297 | 181 | -0.58 | 0.56 |
| housework*stand 10 sec. | .210 | 98 | .258 | 181 | -0.40 | 0.69 |
| housework*transfer indoor | .186 | 98 | .374 | 181 | -1.61 | 0.11 |
| housework*transfer outdoor | .405 | 98 | .203 | 178 | 1.76 | 0.08 |
| housework*move hand to mouth | .017 | 98 | .058 | 181 | -0.32 | 0.75 |
| housework*move hand to head | .035 | 98 | .166 | 180 | -1.04 | 0.30 |
| housework*move hand to back | .138 | 98 | .082 | 180 | 0.45 | 0.66 |
| housework*greeting grip | -.026 | 98 | .140 | 181 | -1.31 | 0.19 |
| housework*pinch grip | -.025 | 98 | .141 | 181 | -1.31 | 0.19 |
|  |  |  |  |  |  |  |
| outdoor mobility*touch big toe | .257 | 96 | .068 | 173 | 1.52 | 0.13 |
| outdoor mobility*stand up | .253 | 98 | .189 | 181 | 0.53 | 0.60 |
| outdoor mobility*stand 10 sec. | .199 | 98 | .235 | 181 | -0.30 | 0.77 |
| outdoor mobility*transfer indoor | .351 | 98 | .221 | 181 | 1.17 | 0.26 |
| outdoor mobility*transfer outdoor | .489 | 98 | .453 | 178 | 0.36 | 0.71 |
| outdoor mobility*move hand to mouth | .119 | 98 | -.056 | 181 | 1.38 | 0.17 |
| outdoor mobility*move hand to head | .190 | 98 | -.051 | 180 | 1.91 | 0.06 |
| outdoor mobility*move hand to back | .139 | 98 | -.103 | 180 | 1.91 | 0.06 |
| outdoor mobility*greeting grip | -.004 | 98 | -.013 | 181 | 0.07 | 0.94 |
| outdoor mobility*pinch grip | .068 | 98 | -.056 | 181 | 0.98 | 0.33 |

Notes: Correlation coefficient group 1= r1 (men); Correlation coefficient group 2= r2 (women); Sample size group 1= n1 (men); Sample size group 2= n2 (women).
