# Supplementary material for: Relationships and gender differences within and between assessments used in Swedish home rehabilitation – a cross-sectional study
Source: BMC Health Serv Res. 2022 Jun 22;22:807. doi: 10.1186/s12913-022-08177-x (PMC9215038; doi:10.1186/s12913-022-08177-x)
Supplement: Supplementary file 2 — Additional file 2. Fischers Z-test between men and women correlation coefficient EQ5D, Sunnaas ADL and GMF. [file 12913_2022_8177_MOESM2_ESM.docx]

Additional file 2. Fischers Z-test between men and women correlation coefficient EQ5D, Sunnaas ADL and GMF

| **EQ5D*Sunnaas ADL** | r1 | n1 | r2 | n2 | z1 | prob.value α |
| --- | --- | --- | --- | --- | --- | --- |
| Mobility*Indoor mobility | .387 | 72 | .410 | 147 | -0.19 | 0.85 |
| Mobility*Outdoor mobility | .100 | 72 | .188 | 147 | -0.61 | 0.54 |
|  |  |  |  |  |  |  |
| Self-care*Dressing/undressing | .574 | 72 | .403 | 147 | 1.55 | 0.12 |
| Self-care*Grooming | .404 | 72 | .339 | 147 | 0.52 | 0.61 |
| Self-care*Bath/shower | .484 | 72 | .381 | 147 | 0.87 | 0.39 |
|  |  |  |  |  |  |  |
| Usual activities*Cooking | .140 | 72 | .339 | 148 | -1.45 | 0.15 |
| Usual activities*Housework | .113 | 72 | .398 | 148 | **-2.10** | **0.04** |
| Usual activities*Outdoor mobility | .014 | 72 | .307 | 148 | **-2.07** | **0.04** |
|  |  |  |  |  |  |  |
| **EQ5D*GMF dependent** |  |  |  |  |  |  |
| Mobility*transfer indoors | .273 | 69 | .249 | 139 | 0.17 | 0.86 |
| Mobility*climb stairs | .359 | 68 | .200 | 131 | 1.14 | 0.26 |
| Mobility*transfer outdoors | .123 | 69 | .009 | 137 | 0.76 | 0.45 |
|  |  |  |  |  |  |  |
| Self-care* touch big toe | .414 | 68 | .173 | 133 | 1.75 | 0.08 |
| Self-care*stand up from sitting position | .307 | 69 | .178 | 139 | 0.92 | 0.36 |
| Self-care*stand more than 10 seconds | .197 | 69 | .243 | 139 | -0.32 | 0.75 |
| Self-care*move hand to mouth | .164 | 69 | .025 | 139 | 0.94 | 0.35 |
| Self-care*move hand to head | .230 | 69 | .041 | 138 | 1.29 | 0.20 |
| Self-care*move hand on back | .289 | 69 | .073 | 138 | 1.49 | 0.14 |
| Self-care*greeting grip | .066 | 69 | .095 | 139 | -0.19 | 0.85 |
| Self-care*pinch grip | .063 | 69 | .099 | 139 | -0.24 | 0.81 |
|  |  |  |  |  |  |  |
| Usual activities* touch big toe | .262 | 68 | .046 | 133 | 1.46 | 0.14 |
| Usual activities*stand up from sitting position | .117 | 69 | .294 | 140 | -1.24 | 0.22 |
| Usual activities*stand more than 10 seconds | .221 | 69 | .269 | 140 | -0.34 | 0.73 |
| Usual activities*transfer indoor | .208 | 69 | .135 | 140 | 0.50 | 0.62 |
| Usual activities*transfer outdoor | .162 | 69 | .202 | 138 | -0.28 | 0.78 |
| Usual activities*move hand to mouth | .080 | 69 | -.133 | 140 | 1.43 | 0.15 |
| Usual activities*move hand to head | .209 | 69 | -.097 | 139 | **2.06** | **0.04** |
| Usual activities*move hand on back | .209 | 69 | -.005 | 139 | 1.44 | 0.15 |
| Usual activities*greeting grip | -.187 | 69 | .099 | 140 | -1.93 | 0.054 |
| Usual activities*pinch grip | .197 | 69 | .090 | 140 | 0.73 | 0.47 |

Notes: Correlation coefficient group 1= r1 (men); Correlation coefficient group 2= r2 (women); Sample size group 1= n1 (men); Sample size group 2= n2 (women).
